# Freestanding HfO_2 _grating fabricated by fast atom beam etching

**DOI:** 10.1186/1556-276X-6-367

**Published:** 2011-04-28

**Authors:** Yongjin Wang, Tong Wu, Yoshiaki Kanamori, Kazuhiro Hane

**Affiliations:** 1 Institute of Communication Technology, Nanjing University of Posts and Telecommunications, Nanjing, Jiang-Su 210003, People's Republic of China; 2 Department of nanomechanics, Tohoku University, Sendai 980-8579, Japan

**Keywords:** HfO_2 _film, grating, fast atom beam etching

## Abstract

We report here the fabrication of freestanding HfO_2 _grating by combining fast atom beam etching (FAB) of HfO_2 _film with dry etching of silicon substrate. HfO_2 _film is deposited onto silicon substrate by electron beam evaporator. The grating patterns are then defined by electron beam lithography and transferred to HfO_2 _film by FAB etching. The silicon substrate beneath the HfO_2 _grating region is removed to make the HfO_2 _grating suspend in space. Period- and polarization-dependent optical responses of fabricated HfO_2 _gratings are experimentally characterized in the reflectance measurements. The simple process is feasible for fabricating freestanding HfO_2 _grating that is a potential candidate for single layer dielectric reflector.

PACS: *73.40.Ty; 42.70.Qs; 81.65.Cf*.

## I. Introduction

As an excellent optical material, hafnium oxide (HfO_2_) film presents high laser damage threshold, thermal and chemical stability [1-3]. Since HfO_2 _film is transparent from visible to infrared range, it often servers as the high refractive index material for fabricating multilayer reflection mirror [4,5], or acts as the waveguiding layer for the realization of guide mode resonant optical filter [6]. These optical devices are originated from the film deposition techniques of HfO_2 _material. On the other hand, freestanding structures are greatly developed as the promising candidates for producing resonant filter [7,8] or in place of a traditional top distributed Bragg reflector to reflect light within a cavity [9-12]. As a single layer dielectric mirror, freestanding structures are often sandwiched with air on top and bottom. Compared with multilayer reflection mirror, freestanding structure is more compact and reflects light more efficiently [13]. The high refractive index contrast between HfO_2_/air also endows the freestanding HfO_2 _micro/nano structures with the capacity to function as single layer dielectric reflector or guide mode resonant filter. HfO_2 _film is a hard material, and usually serves as etch stop layer [14,15]. Recently, focused ion beam (FIB) milling was developed to fabricate sub-micron HfO_2 _gratings [16]. In FIB milling, micro/nano structures could be achieved on various material systems by physically removing the sample material with a metal ion beam. However, FIB milling is a single process and difficult to be compatible with other fabrication processes for mass production. Moreover, this etching technology is expensive and time-consuming.

We demonstrate here a simple way to fabricate freestanding HfO_2 _grating by a combination of fast atom beam (FAB) etching and dry etching of silicon. FAB etching, which is capable of high anisotropy etching because it uses neutral particles or atoms for dry etching, is used as a well-controlled, low-damage etching technique to manufacture HfO_2 _film [17,18]. To make grating structures freely suspend, the silicon substrate beneath the HfO_2 _grating region is removed in association of anisotropic and isotropic dry etching of silicon. Period- and polarization-dependent optical responses are experimentally characterized in reflectance measurements.

## II. Fabrication

**Figure 1:**
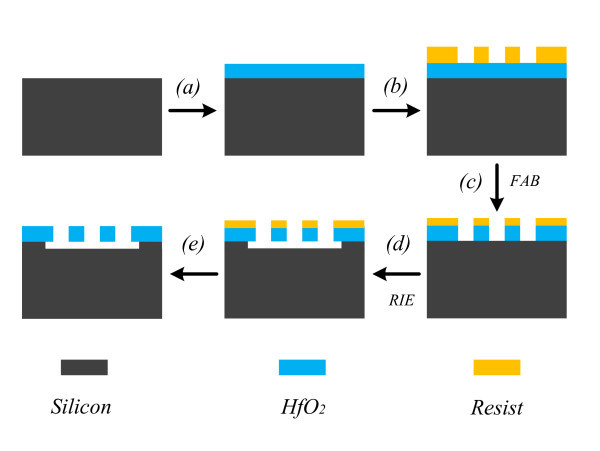
**Fabrication process of freestanding HfO_2 _grating**.

Figure 1 schematically illustrates the fabrication process of freestanding HfO_2 _gratings, which are implemented on a silicon substrate. The process starts from the blank deposition of HfO_2 _film on the silicon substrate with an electron beam (EB) evaporator (step *a*). A positive EB ZEP520A resist is then spin-coated onto the HfO_2 _layer, and grating patterns are patterned in ZEP520A resist using EB lithography (step *b*). Subsequently, the patterns are transferred to HfO_2 _layer by FAB etching (step *c*). FAB etching, which is generated by the neutralization of ions extracted from direct-current SF_6 _plasma (Ebara, FAB-60 ml), is performed with a SF_6 _gas of 5.6 sccm at the high voltage of 2.0 KV and accelerated current of 20 mA. The HfO_2 _gratings are then released by a combination of anisotropic and isotropic dry etching of silicon, which makes the HfO_2 _grating freely suspend (step *d*). The anisotropic etching of silicon is carried out to produce vertical silicon trenches and the isotropic etching is used to release the HfO_2 _gratings laterally, where the remained EB resist and HfO_2 _film act as the etching mask. The freestanding HfO_2 _gratings are finally generated by removing the residual resist (step *e*).

## III. Experimental results and discussion

**Figure 2:**
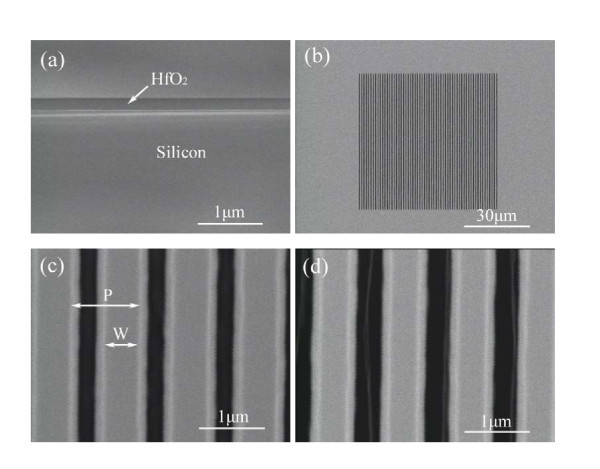
**SEM images of fabricated freestanding HfO**_**2 **_**grating**. (a) cross section SEM image of HfO_2_/Si platform; (b) a fabricated freestanding HfO_2 _grating; (c) and (d) zoom-in SEM images of 1040 nm period HfO_2 _gratings with the grating widths *W*_*t *_of 500 nm and 440 nm, respectively.

Figure 2(a) shows one scanning electron microscope (SEM) image of the cross-section of the HfO_2_/Si platform. The thickness of HfO_2 _film is about 180 nm. The FAB is made up of the energetic neutral beam flux with high directionality and thus, the manufacturing method is capable of high anisotropic etching of HfO_2 _film. There is no special requirement of etching mask, and EB resist can serve as an etching mask. Fabricated freestanding HfO_2 _grating illustrated in Figure 2(b) consists of 60-period grating with the grating length of 60 μm, and air is the low refractive index materials on the bottom and top. The grating period and the grating width are expressed by *P *and *W*. The duty ratio *D*(= *W/P*) is defined as the ratio of the grating width to the grating period. Figures 2(c) and 2(d) illustrate the zoom-in SEM images of the fabricated freestanding HfO_2 _gratings, where the grating period is 1040 nm and the grating height is about 180 nm, the same as the HfO_2 _film thickness. Since the thickness of EB resist varies due to the proximity effect in EB lithography, the HfO_2 _gratings generated in reality are trapezoidal profiles and deviate from the designed rectangular elements. The corresponding bottom grating widths *W*_*b *_are measured ~780 nm and ~670 nm, and the top grating widths *W*_*t *_are about 500 nm and 440 nm, respectively.

**Figure 3:**
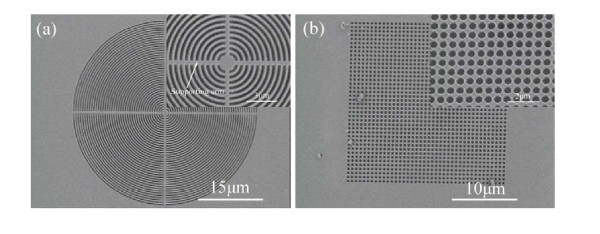
**SEM images of fabricated freestanding HfO**_**2 **_**nanostructures**. (a) SEM image of a freestanding circular HfO_2 _grating, the inset is the zoom-in SEM image of circular grating with the grating period of 500 nm; (b) a freestanding HfO_2 _photonic crystal slab on a GaN-on-silicon platform, the inset is the zoom-in SEM image of HfO_2 _photonic crystals with the grating period of 600 nm.

The simple process is scalable for fabricating suspended HfO_2 _nanostructures, and facilitates monolithic integration of optoelectronic devices on various material systems. Figure 3(a) shows freestanding circular HfO_2 _grating, and the inset is the zoom-in SEM image of circular grating with the grating period of 500 nm, where cross arms are connected to the freestanding circular gratings. From the fabrication point of view, the undercut of silicon beneath the HfO_2 _grating region tends to be difficult when the duty ratio *D *increases. On the other hand, the long HfO_2 _grating beams are in the tendency of being fragile, and the deflection and fracture of HfO_2 _grating beams take place when the duty ratio *D *decreases. According to our experimental results, the duty ratio *D *is feasible in the range of 0.3~0.7 to successfully achieve freestanding HfO_2 _gratings. Moreover, anisotropic and isotropic dry etching of silicon will result in rough silicon surface and large variation in airgap between HfO_2 _grating and silicon beneath HfO_2 _grating region, which will degrade the optical performance. In association of deposition and etching techniques, this fabrication issue can be solved and such freestanding HfO_2 _nanostructures are possible to be incorporated into other material system for serving as the top mirror. Freestanding HfO_2 _photonic crystals illustrated in Figure 3(b) are realized on a GaN-on-silicon platform, and the inset is the zoom-in SEM image of freestanding photonic crystal structures with the period of 600 nm. Between HfO_2 _film and GaN layer, one sacrificial film is inserted. After removing the sacrificial layer, HfO_2 _photonic crystals are freely suspended and the airgap is controlled by the sacrificial layer thickness. These results indicate that the proposed process is feasible to fabricate freestanding HfO_2 _nanostructures.

**Figure 4:**
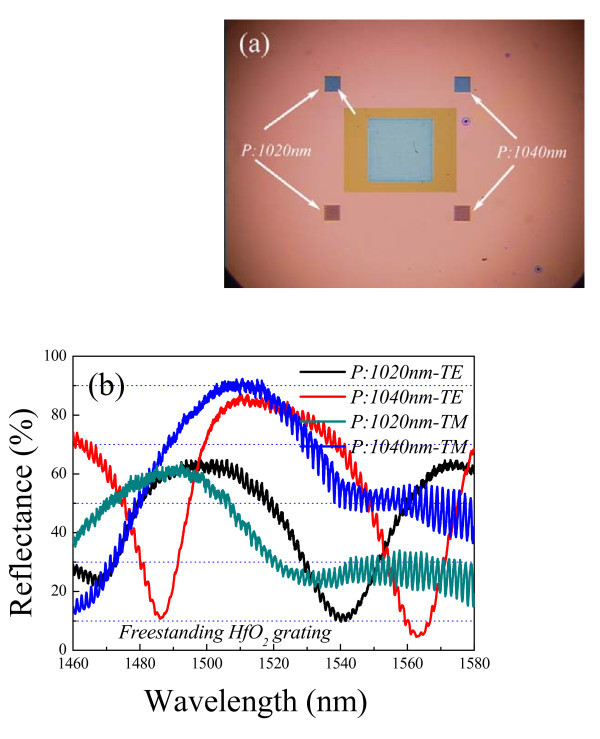
**Optical characterizations of fabricated freestanding HfO**_**2 **_**gratings**. (a) optical micrograph of freestanding HfO_2 _gratings; (b) the reflectance spectra of freestanding HfO_2 _gratings in the telecommunication range.

It should be noted that the HfO_2 _gratings are designed by using rigorous coupled wave analysis (RCWA) method with a commercial code. The generated HfO_2 _gratings deviate much from the ideal elements used for RCWA simulations (not shown here). The trapezoidal grating profiles, roughness of the grating sidewalls, and variations in silicon surface beneath the grating region degrade the optical performance and result in the spectral shift. Moreover, the available spectral range is from 1460 nm to 1580 nm in our measurement system. Hence, a variety of HfO_2 _gratings with different grating parameters are fabricated for optical characterization. Figure 4(a) illustrates one optical micrograph of fabricated HfO_2 _gratings, where the upper two gratings are with the grating widths *W*_*t *_of 440 nm. The color varies as the grating width changes. The grating widths *W*_*t *_are about 500 nm for the bottom gratings, and the grating periods are 1020 nm and 1040 nm, respectively. The inset is the magnified view of fabricated HfO_2 _grating, where the grating period *P *is 1020 nm and the grating width *W*_*t *_is about 440 nm. A tunable laser (Agilent 81682A) is used as the light source to characterize the optical response of the fabricated freestanding HfO_2 _gratings in the telecommunication range. The polarized light beam is incident onto the HfO_2 _gratings by an infrared objective lens with a numerical aperture of 0.25, and an infrared CCD camera is installed on the setup to acquire sample images. The reflected light is collected and sent to an infrared spectrometer. The experimental spectra are normalized to those of a commercial gold mirror. Figure 4(b) illustrates the reflectance spectra of freestanding HfO_2 _gratings, where the grating widths *W*_*t *_are about 440 nm. Taken 1040 nm period HfO_2 _grating as an example, a broad reflection band that is determined by the refractive index contrast is observed under transverse electric (TE) polarization (TE is polarized in the plane of the grating and parallel to the grating lines) [19]. Two sharp reflection dips are found at 1486 nm and 1562.7 nm with measured reflectance of 10.7% and 4.6%, respectively. Measured reflectances are over 70% in the range of 1499.2 m~1539.5 nm. Since fabricated HfO_2 _gratings are configured with one-dimensional symmetry, their optical responses are polarization dependent, which are measured by rotating the sample with an angle of 90° with respect to initial measurement. The reflection band shifts and the shape changes under transverse magnetic (TM) polarization (TM is polarized in the plane of the grating and perpendicular to the grating lines). The linear grating reflector is useful for controlling the polarization on a vertical cavity surface emitting device. A blue-shift is observed in reflectance spectra with decreasing the grating period. As the grating period decreases from 1040 nm to 1020 nm, the broad reflection band shifts to shorter wavelength. These results indicate that freestanding HfO_2 _grating is a promising candidate for single layer dielectric reflector.

## IV. Conclusions

In summary, freestanding HfO_2 _gratings are realized by a combination of FAB etching of HfO_2 _film and dry etching of silicon substrate. Period- and polarization-dependent optical responses of fabricated HfO_2 _gratings are experimentally characterized in the reflectance measurements. The simple process is feasible for fabricating freestanding HfO_2 _grating that is a potential candidate for single layer dielectric reflector.

## Competing interests

The authors declare that they have no competing interests.

## Authors' contributions

YW carried out the device design and fabrication, performed the optical measurements, and drafted the manuscript. TW carried out HfO_2 _film evaporation. YK participated in its design and optical characterization. KH conceived of the study, and participated in its design and coordination. All authors read and approved the final manuscript.
